# The temporal variations in the distribution of filial support from adult children to their aging parents across Europe and Israel

**DOI:** 10.1371/journal.pone.0333873

**Published:** 2025-10-14

**Authors:** Zeynep Zümer Batur, Jeroen K. Vermunt, Dimitri Mortelmans, Jorik Vergauwen

**Affiliations:** 1 Department of Sociology, Centre for Population, Family and Health, University of Antwerp, Antwerp, Belgium; 2 Department of Methodology, Tilburg School of Social and Behavioural Sciences, Tilburg University, Tilburg, Netherlands; Polytechnic Institute of Viana do Castelo, PORTUGAL

## Abstract

The present study aimed to investigate the temporal changes in the support provided by adult children to their ageing parents, including paperwork help, household help, and personal care, and its intensity, ranging from no support to very intense support. We used latent Markov models to assess filial support transitions. Analysing data from the Survey of Health, Ageing and Retirement in Europe (SHARE) covering 13 European countries and Israel between 2004 and 2022, we identified two types of filial support: stable and fluctuating. Our findings indicate that Austria, Germany, the Netherlands, Switzerland, France, Denmark, and the Czech Republic have a higher probability of maintaining stable filial support levels over the years. In contrast, Greece, Belgium, Italy, Spain, Israel, Sweden, and Poland exhibit fluctuating filial support. Additionally, Western and Northern countries, such as Denmark, the Netherlands, Austria, and Germany, tend to reach only moderate or intense support levels. In comparison, Southern and Eastern countries, like Italy, Spain, the Czech Republic, and Poland, provide support that can reach very intense levels.

## Introduction

The focus on informal and formal caregiving for older adults has intensified in European societies, driven by prolonged life expectancy leading to higher demand for long-term care and declining fertility rates limiting the availability of informal caregivers for the ageing population [1]. Many European states provide comprehensive coverage for long-term care while encouraging family members’ involvement in providing informal care for older individuals. In the context of parental care, informal caregiving prevents the institutionalisation of ageing parents, allowing them to remain at home by offering frequent support, such as assistance with paperwork, household tasks, and personal care.

Following the 1990s, European states underwent reforms in providing in-kind and cash benefits to both care recipients and caregivers. These reforms aimed to incentivise informal caregivers to provide greater care while reserving formal care for recipients with severe conditions [2]. Providing informal care is linked to various factors, including the availability of professional care, family size, adherence to traditional family norms, the presence of social insurance, as well as demographic and socioeconomic trends [3].

Among informal caregivers for the ageing population, adult children often serve as the primary caregivers, offering support in the daily activities of their ageing parents, particularly in the absence of a partner. With ongoing demographic trends, it is anticipated that the individual caregiving responsibilities of children will experience a more pronounced increase. [4]. Numerous researchers have observed that daughters exhibit a greater propensity than sons to provide informal care or assume more significant informal caregiving responsibilities for ageing parents [5,6,7,8] and this pattern is particularly prominent in Mediterranean and Eastern European countries compared to Western or Scandinavian countries [9,8]. Despite extensive research on informal care patterns in European countries, much of the existing literature has been cross-sectional [10,11,12] and has not focused on the detailed cross-national differences over time [5,13,14].

Our study aims to further bridge the aforementioned gap in the literature by examining the temporal variations in the intensity of support provided by adult children, analysed on a country-by-country basis. We aim to understand how individual-level filial support and support intensity have changed in European countries. To capture the support and its intensity provided by adult children, we use latent transition analysis (LTA) to accommodate different types of support, including household help, personal care, and assistance with paperwork. Five waves are used, covering 18 years for 14 countries (Germany, Italy, Austria, Sweden, Netherlands, Spain, France, Denmark, Greece, Switzerland, Belgium, Israel, the Czech Republic, and Poland) from the Survey of Health, Aging and Retirement in Europe (SHARE). Our study aims to answer the following research question: How do filial support and its intensity evolve in Europe and Israel over 18 years? By applying latent transition analysis (latent Markov model) in a longitudinal approach, we depict the progression of filial support distribution. This method allows us to thoroughly understand how filial support and its intensity evolve at the individual level over time. Additionally, cross-country comparisons of adult children’s caregiving provide insights into the changing landscape of informal caregiving across Europe.

## Literature review

In research focusing on care for older adults, caregiving typically falls into two primary categories: formal caregiving and informal caregiving. Formal caregiving refers to care services provided by professionals or institutions, such as home healthcare workers, nurses, and residential care facilities. These services are typically regulated, paid, and supported by public or private organizations, and include medical care, personal assistance, and household support [15,2]. In contrast, informal caregiving describes unpaid care provided by family members, friends, or neighbors, encompassing various tasks such as assistance with personal care (e.g., bathing, dressing), financial management (e.g., paperwork), and household chores (e.g., cleaning, shopping). Within informal caregiving, researchers distinguish between general support and intensive caregiving, with the latter involving significant time commitments and demanding activities, particularly assistance with activities of daily living such as feeding, dressing up, and toileting. Intensive caregiving often requires substantial physical and emotional effort and significantly impacts caregivers’ health, employment, and quality of life [16,12].

The extent and intensity of informal caregiving are closely linked to the broader care context—particularly the availability and accessibility of formal long-term care services. Over the past 30 years, European welfare states have implemented various LTC policy changes, such as reducing residential care facilities [17,18], decentralizing social policies in geriatric care [17], outsourcing care services, and introducing competition among commercial care providers [17,19]. Additionally, cash-for-care schemes have emerged, highlighting the growing importance of informal care as policymakers increasingly emphasize its role [20]. Improvements have also been made in the social rights granted to caregivers, such as flexible working hours, protective labour market legislation, and pension recognition for care periods [21].

Researchers exploring the impact of LTC policies on informal caregiving have discussed the “crowding-out” and “crowding-in” theories of the welfare state. The crowding-out theory posits that a generous welfare system can undermine family cohesion; the availability of public pensions and services for older adults may reduce familial solidarity, as children perceive less need to support their parents when the state provides adequate income and services [22]. Conversely, the crowding-in effect suggests that expanding or improving welfare or support services by the state or other formal institutions can increase the willingness or ability of family members to provide care. This is because formal support services, such as financial assistance, respite care, or home health services, can alleviate some of the burdens and stress associated with caregiving, making it more feasible or sustainable for family members to provide care [22,23].

These theories raise the question of whether welfare state services act as substitutes or complements to caregiving. Some researchers suggest that in generous welfare states, informal and formal care serve as substitutes because the state’s services lead to a decline in family contributions [15]. Conversely, other studies indicate that welfare provisions encourage families to care for their aging members by providing financial support and personal care, highlighting the complementary nature of these provisions [16,24,25]. Recent LTC policy reforms suggest that European countries are adopting hybrid policies [20]. These policies encourage individuals to take on caregiving roles (crowding in) by providing support while reducing the necessity for intensive caregiving (crowding out). This approach aims to balance the contributions of the state and the family in providing care.

While these theories and research provide insights into the dynamics of informal and formal care provision within specific contexts, it is essential to recognize that these dynamics vary significantly across countries and the comprehensiveness of their welfare systems. Understanding these differences offers crucial insights into the interplay between individual preferences, cultural norms, and institutional frameworks influencing caregiving practices. In Scandinavia and Western Europe, for example, there is a historically strong emphasis on state responsibility for providing formal care and robust social protection schemes. In contrast, Mediterranean and Southern European countries allocate fewer resources to the needs of their aging populations, resulting in lower levels of formal care provision [26]. In these contexts, religious organizations and community associations have historically played a complementary role in supporting older adults and their families. Particularly in Southern European countries such as Italy, Spain, and Greece, religious institutions—especially the Catholic Church and affiliated charitable groups—have helped fill gaps left by the state, often offering services such as home visits, food aid, and basic care assistance [27,28]. These non-governmental actors not only provide material support but also reinforce familial and community norms around intergenerational care. Although such support is typically less formalized than state provision, it constitutes an important layer in the broader care landscape. These dynamics intersect with broader regional caregiving patterns, which reflect not only differences in state support but also the varying roles of families, communities, and cultural expectations.

Research differentiating between informal care and intensive care illustrates variations in caregiving practices across European countries. Evidence suggests that while Northern countries have more caregivers overall, Southern countries are more engaged in intensive caregiving [12]. Further, studies support the notion that the broader availability of formal care and a strong social welfare system in Nordic and Western European countries contribute to a reduced reliance on informal care among older populations. Conversely, in Southern countries, where family caregiving norms prevail and formal care services are less organized, informal and intensive caregiving methods are more prevalent [16,11,25]. Verbakel et al. [29] also found that countries with a high prevalence of informal caregivers, such as those in the Nordic region, tend to have fewer intensive caregivers. Conversely, Central and Eastern European countries exhibit the opposite pattern. This observation contributes to the discussion on whether welfare states crowd out or crowd in caregiving roles. Generous welfare states appear to encourage people to assume caregiving responsibilities (crowding in) while simultaneously reducing the need for intensive caregiving (crowding out). Verbakel [12] further demonstrated that extensive formal long-term care services reduce the need for intensive caregiving but also motivate more people to offer some level of informal care. Since intensive caregiving is particularly demanding, insufficient formal long-term care services could jeopardize caregivers’ well-being and the sustainability of healthcare systems.

In addition to welfare regimes and cultural expectations, sociodemographic and economic factors play a significant role in shaping caregiving patterns. Research has shown that adult children’s age, gender, and employment status critically influence both the likelihood and intensity of support [14,7,4]. For instance, daughters are more likely than sons to provide care, often taking on more demanding caregiving roles even when employed full-time [14,7]. Additionally, unemployed or part-time workers are generally more involved in intensive caregiving than those with full-time employment, due to greater time availability and flexibility [15,4]. Other relevant factors—such as educational level, household composition, and having dependent children—also influence care provision decisions [16,13]. These individual-level characteristics interact with broader institutional contexts, contributing to cross-national differences in caregiving practices.

Among these factors, gender is particularly central. Across Europe, daughters are more likely than sons to provide informal care to aging parents, and they are also more often responsible for intensive caregiving tasks [6,14,7] Gender norms, expectations of filial duty, and traditional divisions of labor contribute to these disparities and vary in strength across welfare and cultural contexts.

Further, research examining trends in formal and informal caregiving indicates a shift driven by financial constraints, demographic changes, and evolving societal values. One of the most significant shifts was the reduction in the availability of residential care facilities in universalistic systems, such as Nordic countries and the Netherlands. This change aimed to transition from expensive institutional services to more cost-effective home-care options, allowing the elderly to remain in their familiar environments for as long as possible [17,18,30]. In Sweden, for instance, residential care was downsized to accommodate only the most dependent individuals [31]. Additionally, there was a decentralization of social policies in geriatric care, leading to stricter need assessment procedures, the implementation of fee systems as deterrents, and the reorganization of services to limit access to home care [17]. These regulations aimed to promote self-sufficiency among the elderly and prolong independent living in their own homes [19]. For example, in the Netherlands, the long-term care act introduced in 2015 restricted admission to care institutions to those requiring intensive 24-hour supervision [19]. Furthermore, there was a trend toward the externalization of care services in Scandinavian countries, such as Denmark and Sweden, and the introduction of competition among providers to increase productivity [17], resulting in the transfer of care provision from the public to the private sector. Lastly, cash-for-care schemes emerged, emphasizing the importance of informal caregiving as policymakers sought to rely more on family-based support [17]. It is important to highlight that the reduction in services took place primarily at the local level in countries such as the Nordic countries and the Netherlands throughout the 1990s. Most European countries with residual systems expanded their long-term care policies [20]. Differences in caregiving practices across Europe persist despite these trends. Nevertheless, the level of engagement in caregiving may fluctuate based on individual countries and the particular policies regulating formal long-term care.

## Data and method

This study uses data from the Survey of Health, Ageing, and Retirement in Europe (SHARE), offering comprehensive information on the health, socioeconomic status, and social and family networks of individuals aged 50 and older. Since 2004, data has been collected biennially, encompassing most European countries and Israel [32]. The SHARE study undergoes ongoing ethical review. Waves 1–4 were reviewed and approved by the Ethics Committee of the University of Mannheim, while Wave 4 and subsequent phases received approval from the Ethics Council of the Max Planck Society. Additionally, in countries where required, the implementation of SHARE was reviewed and approved by local ethics committees or institutional review boards. These extensive reviews encompassed all aspects of the SHARE study, including sub-projects, confirming compliance with applicable legal standards and alignment with international ethical principles. In addition, participant consent is obtained by interviewers during face-to-face interviews. Participants are informed that they may stop the interview at any time or skip any questions they do not wish to answer. Additionally, they have the option to contact SHARE’s main office if they prefer that their information be removed from the dataset, in which case all associated data are deleted.

Our analysis uses SHARE Waves **[**Waves 3, 4, 5, and 7 were not used because waves 3, 4, 5, and 7 lacked information for at least one variable needed for Markov models. Poland and the Czech Republic were not included in waves 1 and 2, while the Netherlands was absent from wave 6.**]** 1, 2, 6, 8, and 9 (conducted in 2004, 2006, 2015, 2019, and 2022 respectively) to investigate the support that adult children provide to their parents over time across 14 European countries—Austria, Germany, the Netherlands, Switzerland, France, Denmark, the Czech Republic, Greece, Belgium, Italy, Spain, Sweden, Poland, and Israel. The data were accessed on 31/03/2024. In this study, we designate the respondents’ children as support providers and the respondents themselves as support receivers, excluding respondents under the age of 65. After these adjustments, the combined sample comprises 186,071 observation points. The analysis aimed to capture the various forms of filial support that adult children provide to their parents. We applied latent Markov model using Latent GOLD 6.0.

### Forming Markov states

To investigate a complex temporal construct involving various categorical indicators, we utilize latent transition analysis (LTA) with Markov models, employing the Latent GOLD 6.0 software by Vermunt, Tran and Magidson [33]. Latent Markov states are a specialized latent class method for stage-sequential dynamic latent variables, used to predict the probability of transitioning through stages over specific time intervals [34,35]. This approach is the most widely used technique for examining discrete dynamic variables longitudinally [35].

To capture adult children’s support, we used the ‘type of support’ variable. This variable originally has eight categories ((I) no support, (II) paperwork only, (III) household help only, (IV) household and paperwork help, (V) personal care only, (VI) personal care and paperwork (VII) personal care and household help, (VIII) personal care, household and paperwork). In the SHARE survey, personal care includes assistance with activities such as dressing, bathing or showering, eating, getting in or out of bed, and using the toilet. Household help encompasses tasks like home repairs, gardening, transportation, shopping, and household chores. Assistance with paperwork involves activities such as filling out forms and handling financial or legal matters.

To capture the filial support more accurately, we created three binary variables from the original ‘type of support’ variable: paperwork help, household help, and personal care. Coding instances of “paperwork only,” “household only,” and “personal care only” was straightforward. However, when adult children provided multiple types of support, such as both household help and paperwork assistance, we coded ‘yes’ for both the household help and paperwork help binaries, and ‘no’ for the personal care binary variable (cf. Table 1). We primarily use data from waves 1, 2, 6, 8, and 9. For the waves we do not cover (waves 3, 4, 5, and 7), we assigned missing values. This inclusion of missing data was an option in the software program we used. Consequently, the transition probability in our model is always (t + 2) since SHARE data is collected every two years. Thus, the results will indicate the probabilities of changes in filial support over a two-year period. The software accounted for the missing data patterns in its calculations.

**Table 1:** Coding of three binary variables.

Categories	Paperwork	Household	Personal care
No care	0	0	0
Paperwork only	1	0	0
Household help only	0	1	0
Personal care only	0	0	1
Paperwork and household help	1	1	0
Paperwork and personal care	1	0	1
Personal care and household help	0	1	1
Paperwork, household help, and personal care	1	1	1

The goodness of fit statistics, including the Bayesian Information Criterion (BIC) and Akaike Information Criterion (AIC), were calculated for filial support (latent Markov states), as detailed in Table 2. These indices evaluate how well a model fits by balancing its complexity against the sample size [36]. To select the model with the optimal number of states, we primarily relied on the BIC to determine the best-fitting class numbers across countries. When the BIC results were inconclusive, we also reviewed the AIC for the final decision. Latent Markov states range from 2 to 6 (indicating no support to very high intensity) and vary by country.

**Table 2:** Goodness of fit statistics for latent Markov states examining filial support.

Country	Previous Model BIC	Selected Model BIC	Next Model BIC	Optimal Latent Markov States
**Netherlands**	7635.9	**7154.4**	7222.7	**2**
**Switzerland**	7890.3	**7239.4**	7269.2	**2**
**Austria**	17031.8	**16913.9**	16924.7	**3**
**Germany**	18070.1	**17860.9**	17883.5	**3**
**Poland**	10178.9	**10075.3**	10130.3	**4**
**Sweden**	14765.5	**14727.8**	14752.9	**4**
**Spain**	17886.8	**17809.1**	17820.5	**4**
**Italy**	14106.8	**14046.0**	14047.7	**4**
**France**	13677.2	**13635.9**	13689.4	**4**
**Denmark**	12854.0	**12745.2**	12790.2	**4**
**Israel**	13173.4	**13031.5**	13139.6	**4**
**Czech Rep**	30120.1	**30061.5**	30098.1	**4**
**Greece**	22435.1	**22421.8**	22430.6	**6**
**Belgium**	17143.9	**16979.4**	17111.5	**6**

Source: Survey of Health, Ageing and Retirement in Europe (wave 1, wave 2, wave 6, wave 8, and wave 9 release 9.00), calculation by authors

Observations are based on respondents who are 65 or older, N = 186,071

Interpreting these identified states is crucial for understanding filial support patterns across different countries. The “no support” category encompasses adult children who offer no assistance. The “very low” and “low” categories include those who provide minimal assistance, offering at least one type of support. The “moderate” category refers to adult children who provide a moderate level of support, primarily focused on paperwork or household help. The “intensive” and “very intense” categories involve adult children who assist with multiple tasks, including household chores, paperwork, and especially personal care.

## Results

### Descriptive results

The descriptive statistics (cf. Table 3) provide an overview of various variables across five waves collected in 2004 (wave 1), 2006 (wave 2), 2015 (wave 6), 2019 (wave 8), and 2022 (wave 9). The data includes the number of parents and adult children surveyed, varying from 11,781–42,486 and 63,235 and 142,830, respectively. Number of person-period observations are constant at 186,071 for each year.

**Table 3:** Descriptive statistics.

Variables	Years				
	2004	2006	2015	2019	2022
N (parents)	11781	15069	35361	32726	42486
N (adult children)	63235	77844	141759	112901	142830
N (person-period)	186071	186071	186071	186071	186071
***Paperwork (yes)***	4.5%	6.9%	4.7%	4.4%	5.0%
***Household help (yes)***	7.9%	11.4%	9.9%	8.9%	9.3%
***Personal care (yes)***	1.9%	3.5%	2.3%	1.7%	1.7%
***Age of Parents (mean)***	73.7	73.9	74.3	74.8	74.8
***Age of children (mean)***	35.7	36.8	39.6	41.7	41.69
***Gender of parents***					
Female	54.2%	53.9%	54.9%	56.1%	56.7%
Male	45.8%	46.1%	45.1%	43.9%	43.3%
***Gender of adult children***					
Female	49.3%	49.2%	49.4%	49.4%	49.6%
Male	50.7%	50.8%	50.6%	50.6%	50.4%
***Country***					
**Austria**	3170	2562	7368	4347	7415
**Germany**	5751	5774	9232	7872	8808
**Sweden**	7120	6644	9750	6257	6058
**Netherlands**	6808	6332	–	4538	4955
**Spain**	5787	6066	13119	5098	4860
**Italy**	5267	6317	10310	4515	7341
**France**	7141	6870	9293	7003	6635
**Denmark**	2867	6105	8834	5678	5630
**Greece**	5470	6534	9274	5727	5881
**Switzerland**	2091	3204	9145	4604	4067
**Belgium**	8065	7001	12729	5027	9637
**Israel**	8424	8051	6762	4917	2623
**Czech Republic**	–	5472	11220	6954	7463
**Poland**	–	6192	4747	6917	11271

Source: Survey of Health, Ageing and Retirement in Europe (wave 1, wave 2, wave 6, wave 8, and wave 9 release 9.00), calculation by authors

Observations are based on respondents who are 65 or older, N = 186,071

The descriptive results, in Table 3, further demonstrate the percentage of support type provided over 18 years by adult children. Personal care assistance was consistently the least common form of support, with rates generally below 3.5%. Paperwork assistance was also less frequent compared to household help, with percentages ranging from 4.4% to 6.9%. In contrast, household help was the most common type of support, with rates ranging from 7.9% to 11.4%. These general patterns are punctuated by peaks, such as the highest rates of paperwork and household help occurring in 2006, and personal care assistance reaching 3.5% in the same year before declining to 1.7% in the later years. The mean age of parents and children shows a slight increase over the years, with parents averaging around 74.8 years and children around 41.7 years by 2022. The gender distribution of both parents and children remains relatively stable, with a slight increase in the proportion of mothers and a consistent near-equal gender distribution among the children.

The distribution of Markov states (levels of filial support) by country (cf. Table 4) demonstrates the proportion of adult children at different levels of support provided across various countries. In countries with two Markov states, such as the Netherlands and Switzerland, a high percentage of adult children fall into the “no support” category, with 91.81% and 89.12%, respectively, while the remaining percentage provides moderate support.

**Table 4:** Distribution of Markov states by country.

Country	Markov States	No support	Very low	Low	Moderate	Intense	Very intense
Netherlands	2	0.91	―	―	0.08	―	―
Switzerland	2	0.89	―	―	0.10	―	―
Austria	3	0.74	―	―	0.19	0.05	―
Germany	3	0.80	―	―	0.15	0.03	―
Poland	4	0.50	0.42	―	―	0.04	0.01
Sweden	4	0.46	0.41	0.05	―	0.05	―
Spain	4	0.41	0.47	0.04	―	―	0.05
Italy	4	0.87	0.04	0.03	―	―	0.04
France	4	0.64	0.27	―	0.04	0.03	―
Denmark	4	0.70	0.18	―	0.02	0.08	―
Israel	4	0.23	0.31	0.22	―	0.23	―
Czech Rep	4	0.76	―	―	0.08	0.10	0.05
Greece	6	0.38	0.44	0.02	0.02	0.07	0.05
Belgium	6	0.42	0.45	0.02	0.02	0.02	0.03

Source: Survey of Health, Ageing and Retirement in Europe (wave 1, wave 2, wave 6, wave 8, and wave 9 release 9.00), calculation by authors

Observations are based on respondents who are 65 or older, N = 186,071

In Austria and Germany, which have three Markov states, the “no support” category is at 74.52% and 80.95%, respectively. Both countries also display moderate levels of support, with Austria at 19.75% and Germany at 15.92%. The percentage of intense support is smaller, with Austria at 5.73% and Germany at 3.13%.

Countries with four Markov states, such as Poland, Sweden, Spain, Italy, France, Denmark, Israel, and the Czech Republic, demonstrate more diverse support patterns. Italy and France show higher percentages in the “no support” category, with Italy at 87.42% and France at 64.63%, but Italy has small percentages in the “very low” and “low” categories, while France has moderate and intense support levels. Denmark and Israel show significant percentages of adult children in the “no support” state, 70.84% and 23.07% respectively, but Israel has a considerable proportion in the “very low” and “low” categories, and a notable 23.49% in the intense category. The Czech Republic has 76.07% in the “no support” category, with moderate and higher levels of intense support compared to other countries.

Greece and Belgium, which have six Markov states, show even more complexity in their distributions. Greece has 38% in the “no support” category and 44% in the “very low” category, with small percentages across moderate, intense, and very intense states. Similarly, Belgium has 42% in the “no support” state and 45% in the “very low” state, with smaller percentages distributed across the other states.

These distributions highlight the varying degrees of support provided by adult children across different countries, reflecting cultural, social, and possibly policy differences in caregiving responsibilities.

### Transition in filial support in individual European countries over 18 years

We examined how filial support evolves in European countries over 18 years using latent Markov states, based on three types of support: paperwork, household help, and personal care. The number of support states varied across countries: the Netherlands and Switzerland showed two states (no support and moderate support), while Germany and Austria had three (adding intensive support). Countries like Italy, Spain, France, and others showed four states, and Greece and Belgium had the most variation, with six states. In the following section, the transitions in these support patterns across individual countries will be explained.

### Stable filial support

Table 5 presents the transition probabilities of Markov states which shows the probability of filial support change in 2 year-period in the Netherlands and Switzerland, both of which have two states. In the Netherlands, adult children who initially provide no support have a high likelihood (96%) of persisting in this state, with a small probability (3%) of transitioning to moderate support. Conversely, those who start with moderate support have a 66% chance of maintaining this level, and a 33% chance of reverting to no support. In Switzerland, the probabilities are even more skewed towards stability, with a 98% chance of remaining in the no support state and an 89% chance of staying in the moderate support state. These findings suggest that the no support state is particularly stable in both countries, while the moderate support state exhibits more fluctuation, especially in the Netherlands.

**Table 5:** Transition of filial support by countries with two Markov states.

Netherlands	States	No support	Moderate
**No support**	0.96	0.03
**Moderate**	0.33	0.66
**Switzerland**	States	**No support**	**Moderate**
	**No support**	0.98	0.01
	**Moderate**	0.10	0.89

Source: Survey of Health, Ageing and Retirement in Europe (wave 1, wave 2, wave 6, wave 8, and wave 9 release 9.00), calculation by authors

Observations are based on respondents who are 65 or older, N = 186,071

Table 6 illustrates the transition probabilities of Markov states in Austria and Germany, each having three states. In Austria, the probabilities of remaining in the initial state are quite high across all levels of support: 95% for no support, 96% for moderate support, and 78% for intense support. This indicates a general stability in filial support levels over a two-year period, with the most stability observed in the moderate support state. The intense support state, however, shows some tendency towards no support. In Germany, the probabilities of remaining in the initial state are similarly high: 98% for no support, 97% for moderate support, and 69% for intense support. This suggests that filial support levels in Germany are also quite stable, particularly in the no support and moderate support states. However, the intense support state does show some propensity to transition to no support.

**Table 6:** Transition of filial support by countries with three Markov states.

Austria	States	No support	Moderate	Intense
**No support**	0.95	0.02	0.01
**Moderate**	0.002	0.96	0.03
**Intense**	0.19	0.01	0.78
**Germany**	States	**No support**	**Moderate**	**Intense**
	**No support**	0.98	0.006	0.004
	**Moderate**	0.01	0.97	0.01
	**Intense**	0.30	0.005	0.69

Source: Survey of Health, Ageing and Retirement in Europe (wave 1, wave 2, wave 6, wave 8, and wave 9 release 9.00), calculation by authors

Observations are based on respondents who are 65 or older, N = 186,071

Moreover, Table 7 reveals a stable pattern of filial support levels over a two-year span in France, Denmark, and the Czech Republic. In France and Denmark, for example, adult children who initially provide no support, moderate support, or intense support are highly likely to maintain these states. Specifically, the probabilities are 70%, 90%, and 77% in France, and 88%, 88%, and 86% in Denmark, respectively. Meanwhile, the Czech Republic also demonstrates stability across the Markov states, with probabilities of 94% for no support, 69% for moderate, 86% for intense, and 76% for very intense. It is noteworthy that the Czech Republic exhibits high-level filial support states, indicating the absence of very low or low states.

**Table 7:** Transition of filial support by countries with four Markov states.

Italy	States	No support	Very low	Low	Very intense
**No support**	0.99	0	0	0
**Very low**	0.02	0.23	0	0.74
**Low**	0	0.71	0.27	0
**Very intense**	0.08	0	0.60	0.31
**Spain**	States	**No support**	**Very low**	**Low**	**Very intense**
	**No support**	0	0.97	0	0.02
	**Very low**	0.99	0	0	0
	**Low**	0	0.15	0	0.84
	**Very intense**	0	0	0.80	0.19
**France**	States	**No support**	**Very low**	**Moderate**	**Intense**
	**No support**	0.70	0.29	0	0
	**Very low**	0.94	0	0.03	0.02
	**Moderate**	0.02	0.06	0.90	0
	**Intense**	0.15	0.07	0	0.77
**Denmark**	States	**No support**	**Very low**	**Moderate**	**Intense**
	**No support**	0.88	0.11	0	0
	**Very low**	0.90	0	0.02	0.07
	**Moderate**	0.10	0.03	0.86	0
	**Intense**	0	0.10	0	0.88
**Sweden**	States	**No support**	**Very low**	**Low**	**Intense**
	**No support**	0.23	0.76	0	0
	**Very low**	0.98	0	0.01	0
	**Low**	0	0.04	0	0.94
	**Intense**	0.01	0	0.92	0.04
**Israel**	States	**No support**	**Very low**	**Low**	**Intense**
	**No support**	0	0	0	0.99
	**Very low**	0.99	0	0	0
	**Low**	0	0.98	0	0.01
	**Intense**	0	0	0.98	0.01
**Czech Republic**	States	**No support**	**Moderate**	**Intense**	**Very intense**
	**No support**	0.94	0.04	0	0
	**Moderate**	0.30	0.69	0	0
	**Intense**	0.09	0.02	0.86	0.01
	**Very intense**	0.23	0	0	0.76
**Poland**	States	**No support**	**Very low**	**Intense**	**Very intense**
	**No support**	0.26	0.70	0	0.02
	**Very low**	0.99	0	0	0
	**Intense**	0.11	0.01	0.86	0
	**Very intense**	0.07	0.73	0	0.18

Source: Survey of Health, Ageing and Retirement in Europe (wave 1, wave 2, wave 6, wave 8, and wave 9 release 9.00), calculation by authors

Observations are based on respondents who are 65 or older, N = 186,071

It is crucial to highlight that the categorization process is based on examining the overall transitions, or stability, from one state to another. The countries mentioned earlier demonstrate stability across all or most of the Markov states. Following this, we will identify the countries that do not exhibit overall stability across the states, but rather display more fluctuation in filial support states.

### Fluctuation in filial support

Countries such as Italy, Spain, Sweden, Israel, Poland (cf. Table 7), Greece, and Belgium (cf. Table 8) display fluctuations in filial support to their ageing parents. For these countries, the shifts in proportions suggest more dynamic transitions. When interpreting fluctuating Markov states, we will specifically focus on major transitions (fluctuations) such as from very intense support to low or very low support, or vice versa. Transitions like from no support to very low support do not represent a substantial change in filial task uptake.

**Table 8:** Transition of filial support by countries with six Markov states.

Greece	States	No support	Very low	Low	Moderate	Intense	Very intense
**No support**	0	0.88	0	0	0.11	0
**Very low**	0.99	0	0	0	0	0
**Low**	0	0	0	0.19	0	0.80
**Moderate**	0	0.12	0	0.83	0.03	0
**Intense**	0.17	0.54	0	0	0.27	0
**Very intense**	0	0	0.15	0	0.13	0.71
**Belgium**	States	**No support**	**Very low**	**Low**	**Moderate**	**Intense**	**Very intensive**
	**No support**	0.10	0.89	0	0	0	0
	**Very low**	0.98	0	0	0	0	0.01
	**Low**	0	0	0	0.98	0	0.01
	**Moderate**	0	0	0	0	0.01	0.98
	**Intense**	0	0	0.97	0	0	0.02
	**Very intensive**	0	0.15	0	0	0.82	0

Source: Survey of Health, Ageing and Retirement in Europe (wave 1, wave 2, wave 6, wave 8, and wave 9 release 9.00), calculation by authors

Observations are based on respondents who are 65 or older, N = 186,071

Looking at Italy, we see that adult children providing very intense support have a 60% chance of transitioning to low support. Those offering very low support are most likely to transition to a state of very intense support, with a probability of 0.74. In Spain, individuals with very intense support have a high probability of transitioning to low support (80%), and those with low support are most likely to move to very intense support (84%).

For Sweden, Israel, and Poland, we observed similar patterns of filial support transitions as in Italy and Spain. In Sweden, individuals with low support are highly likely to transition to intense support (94%), while those with intense support have a high probability (92%) of transitioning to low support. In Israel, the chances are 99% for adult children to transition from giving no support to giving intense support within a two-year period. The transition from intense to low support also has a similar probability (98%). In Poland, we observe a one-direction transition from very intense filial support to very low support, with a 73% probability.

Belgium and Greece have the widest range of filial support states and the most fluctuations, especially Belgium. In Greece, those with low support are most likely to transition to very intense support (80%), and adult children with intense support have a 54% chance of transitioning to very low support. In Belgium, adult children with low support have a very high probability (98%) of transitioning to moderate support. Those with intense support are highly likely (97%) to transition to low support. For adult children providing moderate support in Belgium, they are most likely to transition to a state of very intense support, with a probability of 98%.

These findings illustrate the dynamic nature of filial support across different countries, highlighting both common patterns and unique variations in how adult children adjust their support levels over time. Understanding these transitions can help inform policies and interventions aimed at supporting ageing parents and their families more effectively.

## Discussion

As Europe’s population ages, the demand for geriatric care is rising. Factors like longer life expectancy, changes in care policies, higher female labour force participation, smaller family sizes, and shifts in family structures have led researchers to study informal caregiving by adult children [37,13,38]. Studies demonstrated that care provision varies across Europe due to differences in welfare systems and cultural norms. Scandinavia and Western Europe emphasize state responsibility for formal care, while Mediterranean and Southern Europe provide fewer resources, leading to more informal care [26]. Northern countries have more caregivers overall, while southern countries rely more on intensive family caregiving due to less organized formal services [16,11,25,12].

Our study examines variations in filial support across 13 European countries and Israel. By using different types of support to capture the evolution of filial support transitions, we applied latent Markov model and identified six types of states for adult children: ‘no support,’ ‘very low,’ ‘low,’ ‘moderate,’ ‘intense,’ and ‘very intense.’ Latent Markov model allowed us to observe the probabilities of transitioning from one state to another over a two-year period.

The distribution of Latent Markov States across various countries, as presented in Table 4, highlights some significant differences in support levels that warrant attention. For example, the ‘very intense’ support state is missing in many Western and Northern countries, with Belgium as an exception. Additionally, the ‘low’ filial support level is generally absent in these countries, except for Sweden and Belgium. These patterns underscore the impact of national welfare systems and cultural norms on caregiving practices. In countries with more generous welfare systems—particularly in Northern and Western Europe—formal care services are widely available and accessible. This might reduce the necessity for family members to provide very intense care and supports more stable, moderate levels of informal support. In contrast, in Southern and Eastern European countries, formal care systems are often less extensive, and cultural norms place a stronger emphasis on family responsibility in old age. These normative expectations, combined with limited formal alternatives, can help explain both the higher prevalence of intense support and the greater fluctuations in care observed in these contexts.

Moreover, in our analysis of 14 countries, we identified two patterns of overall transitions in filial support: stable and fluctuating. Stable filial support indicates that the support level remains consistent over two years, while fluctuating support means there is a change from one support state to another during the same period. Countries with stable filial support include Austria, Germany, Switzerland, the Netherlands, France, Denmark, and the Czech Republic. It is crucial to note that, except for the Czech Republic, these countries do not have adult children providing ‘very intense’ care.

In contrast, the countries displaying fluctuations between states—Greece, Belgium, Sweden, Italy, Spain, Israel, and Poland—show a high probability of changes in filial support over a two-year period. Sweden stands out in the list as it does not align with this group and the Northern-Southern classification made in the literature. The fluctuating levels of filial support observed in Sweden can be attributed to recent developments in social policy, where the need for public support of informal caregivers has gained prominence in several countries. In response to the strain on formal care systems, countries like Sweden have increasingly recognized the importance of informal caregiving, integrating it into the broader framework of care provision [39]. Spain, Italy, and Poland have adult children providing ‘very intense’ filial support, whereas this level of support is not observed in Israel and Sweden.

Setting aside overall movement and focusing on stability within specific Markov states Austria, Germany, France, and Denmark consistently maintain high percentages of stable moderate and intense support states over the years. In Greece, adult children provided stable filial support at both moderate and very intense levels. The Netherlands and Switzerland showed stability in moderate support, while Poland demonstrated stability in intense support. The Czech Republic exhibited the most stability across higher levels of filial support, including moderate, intense, and very intense states.

As mentioned above and in the literature, there is a difference in the intensity between Northern and Southern countries. We also found that Southern and Eastern countries, i.e., Greece, Spain, Italy, Poland, and Czech Republic have adult children who provide intensive support while it is not the case for the Northern and Western countries, i.e., Germany, Austria, Denmark, the Netherlands. However, we observed that the probability of fluctuating filial support is higher in the Southern and Eastern countries and lower in the Northern and the Western. One of the reasons for these shifts in Southern countries might lie in their formal care provisions. Northern countries offer more comprehensive formal care to their ageing population and their informal caregivers, enabling a stable level of filial support. However, in the absence of adequate formal support for informal caregivers, such as adult children, individuals in Southern countries might frequently shift between different levels of support. Since maintaining very intensive support is challenging, fluctuations in support intensity occur. In contrast, in northern countries with robust formal care policies, informal caregivers are less burdened with providing very intensive care, allowing them to maintain stable levels of support.

While country-level welfare systems and cultural norms help explain many of the cross-national differences observed in our study, some fluctuations in filial support likely stem from individual-level changes in the adult children’s own circumstances. For instance, transitions such as job loss, retirement, changes in health status, or shifts in household composition may influence an adult child’s capacity to provide care at a given time. These micro-level dynamics are not directly captured in our latent Markov model but could be critical in understanding the more fluid care trajectories observed in certain contexts. Incorporating such variables in future research could help disentangle the relative impact of institutional versus life-course factors in shaping caregiving transitions.

Although latent transition analysis and Markov models are powerful statistical techniques and are well-suited for our study, they do have certain limitations. One significant limitation, particularly for our study, is the interpretation of latent states. These states can sometimes be challenging to interpret substantively, and the labels and meanings assigned to them require careful consideration and justification, which we have meticulously addressed.

This work is crucial for examining country-specific, longitudinal variations in filial support. We captured the majority of tasks that adult children can provide to their ageing parents and covered a wide range of support possibilities (from no support to very intense support). This study serves as a first step toward a more detailed investigation of changes in filial support. Future research should explore why some countries exhibit stable filial tasks over the years while others fluctuate. Additionally, further studies could incorporate a gender perspective and other demographic characteristics, such as labour force participation, educational status, having children, and marital status, which play significant roles in the lives of adult children. Overall, the results contribute to our understanding of the evolving landscape of filial support, taking into account country-specific differences.
